# Right hemicolectomy with complete mesocolic excision is safe, leads to an increased lymph node yield and to increased survival: results of a systematic review and meta-analysis

**DOI:** 10.1007/s10151-021-02471-2

**Published:** 2021-06-12

**Authors:** G. Anania, R. J. Davies, F. Bagolini, N. Vettoretto, J. Randolph, R. Cirocchi, A. Donini

**Affiliations:** 1 Dipartimento di Scienze Mediche, Università degli Studi di Ferrara, Ferrara, Italy; 2 Cambridge Colorectal Unit, Addenbrooke’s Hospital, Cambridge University Hospitals NHS Foundation Trust, Cambridge, UK; 3 Montichiari Surgery, ASST Spedali Civili, Brescia, Italy; 4 Georgia Baptist College of Nursing. Mercer University, Atlanta, GA USA; 5 Department of Medicine and Surgery, University of Perugia, Perugia, Italy; 6 Azienda Ospedaliera Di Terni, 05100 Terni, Italy

**Keywords:** CME, Complete mesocolic excision, Safety, Lymph node yield, Meta-analysis, Colon cancer

## Abstract

**Background:**

The introduction of complete mesocolic excision (CME) for right colon cancer has raised an important discussion in relation to the extent of colic and mesenteric resection, and the impact this may have on lymph node yield. As uncertainty remains regarding the usefulness of and indications for right hemicolectomy with CME and the benefits of CME compared with a traditional approach, the purpose of this meta-analysis is to compare the two procedures in terms of safety, lymph node yield and oncological outcome.

**Methods:**

We performed a systematic review of the literature from 2009 up to March 15th, 2020 according to the Preferred Reporting Items for Systematic Reviews and Meta-Analyses (PRISMA) guidelines. Two hundred eighty-one publications were evaluated, and 17 met the inclusion criteria and were included. Primary endpoints analysed were anastomotic leak rate, blood loss, number of harvested lymph nodes, 3- and 5-year oncologic outcomes. Secondary outcomes were operating time, conversion, intraoperative complications, reoperation rate, overall and Clavien–Dindo grade 3–4 postoperative complications.

**Results:**

In terms of safety, right hemicolectomy with CME is not inferior to the standard procedure when comparing rates of anastomotic leak (RR 0.82, 95% CI 0.38–1.79), blood loss (MD −32.48, 95% CI −98.54 to −33.58), overall postoperative complications (RR 0.82, 95% CI 0.67–1.00), Clavien–Dindo grade III–IV postoperative complications (RR 1.36, 95% CI 0.82–2.28) and reoperation rate (RR 0.65, 95% CI 0.26–1.75). Traditional surgery is associated with a shorter operating time (MD 16.43, 95% CI 4.27–28.60) and lower conversion from laparoscopic to open approach (RR 1.72, 95% CI 1.00–2.96). In terms of oncologic outcomes, right hemicolectomy with CME leads to a higher lymph node yield than traditional surgery (MD 7.05, 95% CI 4.06–10.04). Results of statistical analysis comparing 3-year overall survival and 5-year disease-free survival were better in the CME group, RR 0.42, 95% CI 0.27–0.66 and RR 0.36, 95% CI 0.17–0.56, respectively.

**Conclusions:**

Right hemicolectomy with CME is not inferior to traditional surgery in terms of safety and has a greater lymph node yield when compared with traditional surgery. Moreover, right-sided CME is associated with better overall and disease-free survival.

## Introduction

Tumors of the colon and rectum are the second most common tumor in women and the third in men [1]. Cancers located in the right colon, left colon and rectum appear to be different entities, and evolve differently. Surgery remains the mainstay of treatment when potential for cure is the aim. While surgical techniques for the rectum [2] have now been largely standardized and can be performed by different modes of access, there is ongoing debate about the extent of colic and mesenteric resection in surgery on the right colon and radicality of lymph node excision. Building on the concept of total mesorectal excision (TME), a new surgical era has opened for the right colon.

Complete mesocolic excision (CME) for the right colon was first described in 2009 by the Erlangen group (Germany) [3], and subsequently, a similar concept referred to as D3 lymphadenectomy was reported in Asia. These ideas of an extended resection with potential increased oncological radicality became topics of great interest to surgeons worldwide [4]. It is a surgical procedure that involves the complete separation of the parietal and visceral embryological planes, extending the resection to include the pancreatic lymph node stations and in some cases up to the greater curvature of the stomach [3]. This is associated with ligation at the origin of the appropriate colic vessels (known as central vascular ligation [CVL]), to widen the lymphatic resection and extension of the primary intestinal resection proportionate to the staging of the tumour.

There is still uncertainty about the indications for CME and the advantages of the technique compared to traditional right hemicolectomy [6] and CME is being evaluated in numerous prospective studies. The purpose of this review and meta-analysis was to analyze the available data on right hemicolectomy with CME vs. traditional right hemicolectomy in terms of safety, feasibility and oncological outcomes, and attempt to define the role of this controversial surgical procedure.

## Materials and methods

We performed a systematic review of the literature from inception up to March 15th, 2020 according to the Preferred Reporting Items for Systematic Reviews and Meta-Analyses (PRISMA) guidelines [4]. Randomised controlled trials (RCTs) and non-randomised controlled trials, which compared CME vs non-CME right hemicolectomy for colon cancer were considered for inclusion regardless of the surgical approach or the outcomes reported; if we had found both types of studies (RCTs and non-RCTs), we would have had to perform two separate meta-analyses and not a single meta-analysis.

All non-comparative studies were excluded. In the case of patients overlapping between two or more studies, only the most recent study was considered.

The comprehensive search of the literature was performed by analysing the relevant databases: Medline/PubMed, Scopus, Web of Science, CNKI (中国知网) (China National Knowledge Infrastructure) Wanfang Data (万方) and other sources (Google Scholar) for articles reporting data on CME vs non-CME right hemicolectomy, without any language restrictions. The references of all included studies were screened to identify any study missed during the initial search.

The following search statement was used in Medline/PubMed:

- cme[All Fields] AND right[All Fields] AND ("colectomy"[MeSH Terms] OR "colectomy"[All Fields])
- complete[All Fields] AND mesocolic[All Fields] AND excision[All Fields] AND right[All Fields] AND ("colectomy"[MeSH Terms] OR "colectomy"[All Fields])
- cme[All Fields] AND right[All Fields] AND ("colectomy"[MeSH Terms] OR "colectomy"[All Fields] OR "hemicolectomy"[All Fields])
- complete[All Fields] AND mesocolic[All Fields] AND excision[All Fields] AND right[All Fields] AND ("colectomy"[MeSH Terms] OR "colectomy"[All Fields] OR "hemicolectomy"[All Fields])

In the other bibliographic databases (WOS, Scopus, CNKI and Wanfang Data), the search was performed by entering the association of the following keywords:

- cme AND right AND colectomy
- cme right hemicolectomy
- complete AND mesocolic AND excision AND right AND colectomy
- complete AND mesocolic AND excision AND right AND hemicolectomy

Successively, another search was performed through the reference lists of the selected articles and relevant grey literature through Google Scholar. The studies of each database were included in the bibliographic software package and the duplicate records were excluded. Furthermore, ClinicalTrials.gov was searched to collect the registered ongoing clinical trials. Two authors (RC, AG) individually evaluated the titles and abstracts of all studies. The full text of studies that could potentially fulfil the inclusion criteria was obtained. The same two authors independently assessed these full texts to determine whether they met the inclusion criteria for this review.

Successively, the same two authors (RC, AG) individually extracted data from the studies. The information collected from each study was as follows: year of publication, study design, inclusion criteria, exclusion criteria, and outcomes.

The primary outcomes were the anastomotic leak rate, the estimated blood loss, the overall number of harvested lymph nodes and 3-year to 5-year oncologic outcomes.. The secondary outcomes were the operative time, the conversion from laparoscopy to open right hemicolectomy, the intraoperative complications (e.g. vascular injuries, iatrogenic small bowel perforation), the overall postoperative complications, the Clavien–Dindo grade III–IV [5] postoperative complications and the reoperation rate. Robotic, laparoscopic and open cases were included in the analysis.

### Statistical analysis

We calculated risk ratios (RR) for dichotomous variables and weighted mean differences (WMD) for continuous variables. Intention-to-treat analysis was performed.

The Mantel–Haenszel method was used for the meta-analysis. All results were displayed in a forest plot graph. The *Q* test was used to analyze the heterogeneity. An *I*^2^ statistic value ≥ 75% indicates a considerable level of heterogeneity. The data analysis was performed using the meta-analysis software Review Manager (RevMan) v 5.3.5 (Copenhagen: The Nordic Cochrane Centre, The Cochrane Collaboration, 2018)[6].

The Risk of Bias In Non-randomized Studies of Interventions (ROBINS-I) assessment tool was used to evaluate the methodological quality of the included studies [7], graphic visualization of the results was obtained with the aid of the ROBINS online tool [8]. All studies begin with the assumption of a low level of risk of bias and were then downgraded by one or two ROBINS-I levels based on the applicable domains in the ROBINS-I tool.

Among all primary outcomes, subgroup analysis for different populations was performed between Asia and Europe.

We assessed the overall quality of the evidence for each outcome according to the Grading of Recommendations Assessment, Development and Evaluation (GRADE) approach [9]. We initially downgraded studies up to two levels in the GRADE system based upon the degree of risk of bias assessed using the ROBINS-I tool. We then downgraded the certainty of evidence further based on the domains specified in the GRADE system.

The protocol for this systematic review and meta-analysis was submitted and accepted from PROSPERO: CRD42020166049 (http://www.crd.york.ac.uk/prospero).

## Results

**Fig. 1:**
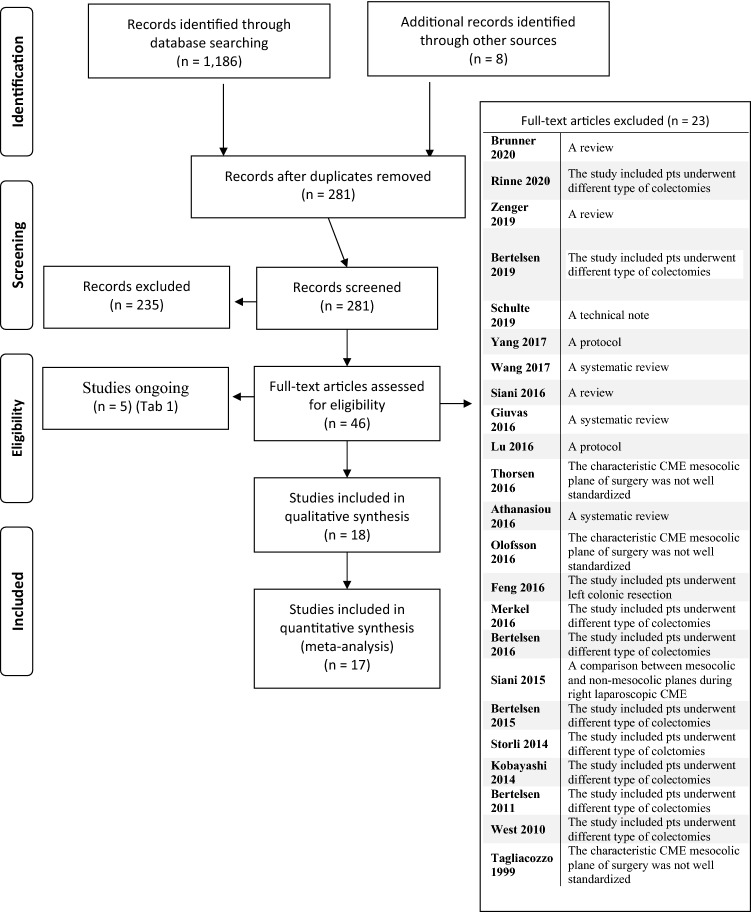
Prisma flowchart of literature search

We retrieved 1194 records with our search strategy (Fig. 1). Among these, 913 were excluded, because they were duplicated. Subsequently, 281 titles and abstracts were evaluated, with 235 abstracts excluded, because they did not meet the inclusion criteria. After the evaluation of 46 full texts, 23 articles were excluded [10–32] with five ongoing studies (Table 1). Eighteen non-randomised controlled trials were included in the qualitative analysis, and 1 study [33] was subsequently excluded from quantitative synthesis, because the reported data were not adequate. Therefore, 17 were included in the meta-analysis (Table 2) [34–50].

**Table 1:** Ongoing studies

Name of study	Type of study	Year of registration	Registration	Countries of recruitment	Trial participating centre	Estimate number patients to enrol	Intervention	Control
REK 2015/2396	RCT	2018	NCT0377659	Norway	NR	218	Laparoscopic CME right colectomy	Open D3 right colectomy
RESECTAT trial	Prospective open registry-based	2017	NR	Germany	39	1000	CME right colectomy	Standard right colectomy
COMET	Multi-centre cluster RCT	2016	ISRCTN45051056	UK	4	80	CME right colectomy	Standard right colectomy
RELARC	RCT	2016	NCT02619942	China	17	1072	Laparoscopic CME right colectomy	D2 dissection laparoscopic right colectomy
SLRC	RCT	2016	NCT02942238	China	NR	582	Laparoscopic CME right colectomy	Open D3 right colectomy

*RCT* randomized controlled trial, *RESECTAT* CME or traditional surgery for right-sided colon cancer. Protocol of a registry-based multicenter prospective non-randomized trial, *REK 2015/2396* open D3 right colectomy compared to laparoscopic CME right colectomy for right-sided colon cancer; an Open Randomized Controlled Study, *COMET* complete mesocolic excision vs. standard of care right hemicolectomy randomised controlled Trial, *RELARC* radical extent of lymphadenectomy—D2 dissection versus complete mesocolic excision of LAparoscopic Right Colectomy for right-sided colon cancer, *SLRC* standardization of laparoscopic surgery for right hemi colon cancer, *UK* United Kingdom, *CME* complete mesocolic excision

**Table 2:** Inclusion criteria

Author—year of publication	Nation	Type of study	N. of patients included	Time of enrolment	Type of access
Pedrazzani 2020 [43]	Italy	R	114	2014–2019	LA
Yozgatli 2019 [48]	Turkey	P	96	2015–2017	RA/LA
Ho 2019 [39]	Singapore	R	25	2012–2015	LA
Ouyang 2019 [42]	China	R	167	2008–2015	LA
Zurleni 2018 [50]	Italy	R	192	2007–2012	OA
Prevost 2018 [44]	Switzerland	R	155	2001–2015	LA/OA
An 2018 [34]	South Korea	R	115	2007–2011	LA
Bertelsen 2018 [35]	Denmark	R	465	2008–2014	LA/OA
Cao 2018 [36]	China	R	189	2006–2017	LA
Zhao 2017 [49]	China	R	47	2010–2015	LA/OA
Yang 2017 [23]	China	R	125	2012–2015	LA/OA
Lieto 2017 [40]	Italy/Egypt	R	134	2008–2016	OA
Procházka 2016 [45]	Czech Republic	P	83	2014–2015	OA
Qin 2016 [46]	China	R	336	2005–2014	OA
Liu 2015 [41]	China	R	70	2010–2014	LA
Galizia 2014 [37]	Italy	P	103	2008–2012	OA
Gao 2012 [38]	China	R	92	2008–2011	OA

*RCT* randomized controlled trial, *R* observational retrospective, *P* observational prospective, *LA* laparoscopic assisted, *RA* robotic assisted, *OA* open access

### Description of studies.

A detailed description of the 17 included studies, and patient characteristics is provided in Table 2: the researchers enrolled 2508 patients (1203 CME and 1305 non-CME). Ten studies were performed in Asia (1262 patients, 50.32%), with China the nation performing the highest number of studies (7 studies, 1026 patients; 40.90%). The other 7 studies were performed in Europe (1246 patients, 49.68%), with Italy, the European nation performing the highest number of studies (543 patients, 21.29%). One of these Italian studies was performed in collaboration with colleagues from Egypt [40]. The studies included were published between 2012 and 2020; the patients were enrolled between 2001 and 2019. There were no significant differences in age, sex, body mass index, American Society of Anesthesiologists class or TNM stage between the CME and non-CME groups.

### Assessment of risk of bias in included studies

The risk of bias in the included studies was independently assessed by two authors (RC, FB).

**Fig. 2:**
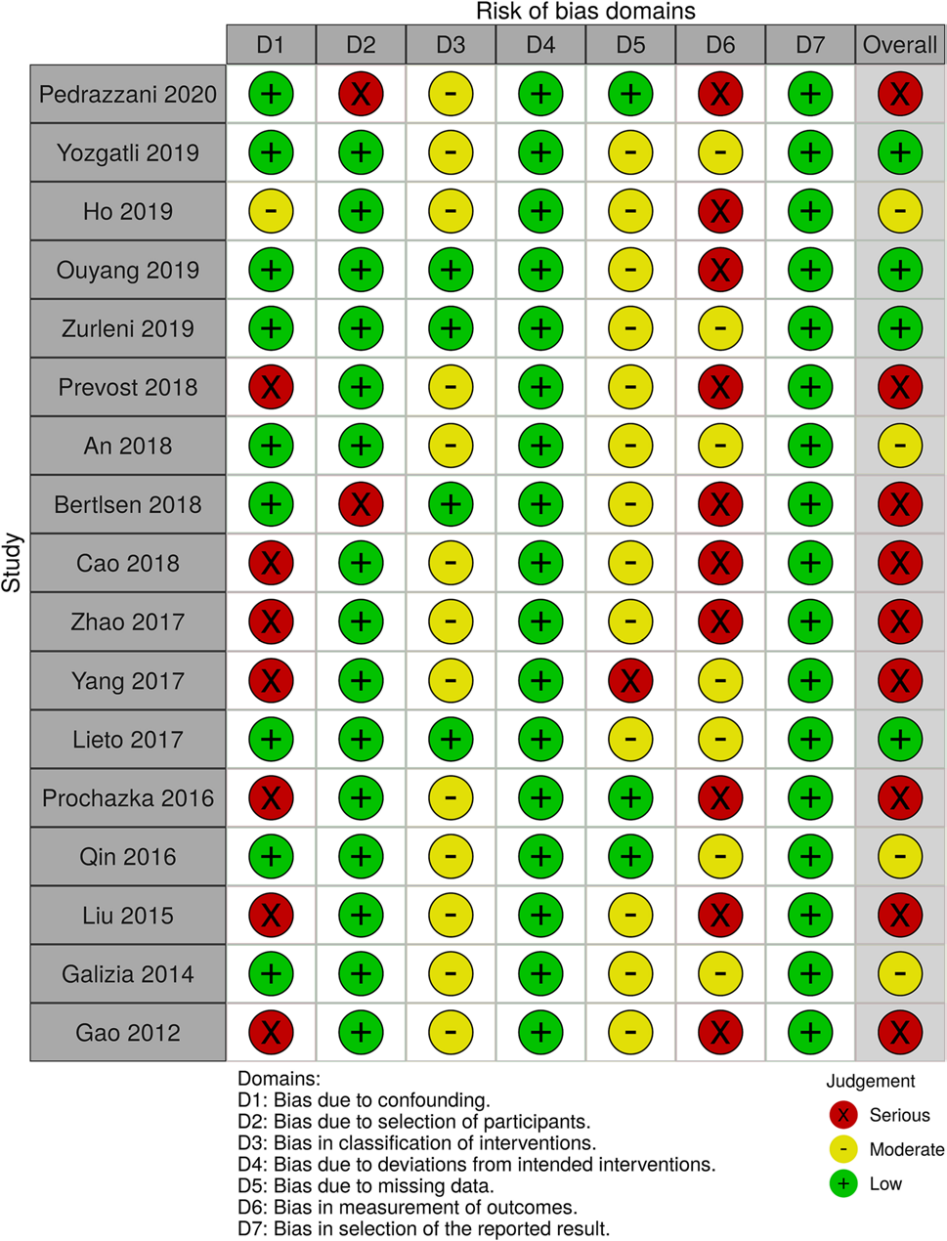
The risk of bias according to ROBINS-I tool

In the ROBINS-I tool, risk-of-bias judgments may be classified as *low, moderate, serious* or *critical*. Four out of 17 studies were assessed as having low risk of overall bias, while 4 were determined as having moderate risk, and 9 as having serious risk. The most common cause of serious risk of bias was confounding. Concerning the domain of selection bias regarding study participants, 15 studies were evaluated to be at low risk of bias. Regarding bias in classification of the interventions, four were deemed to have low risk of bias and the rest were deemed to have moderate bias. Bias due to deviations from intended interventions was low in all studies. The evaluation of missing data bias was deemed as low risk in three studies; the other studies were deemed to have moderate risk with the exception of one study with serious risk. Regarding bias in selection of the measurement of the outcomes, the studies all had a low risk of bias. In terms of bias of reported results, four studies were at low risk or moderate risk; the rest of the studies were at high risk of bias (Fig. 2).

**Fig. 3:**
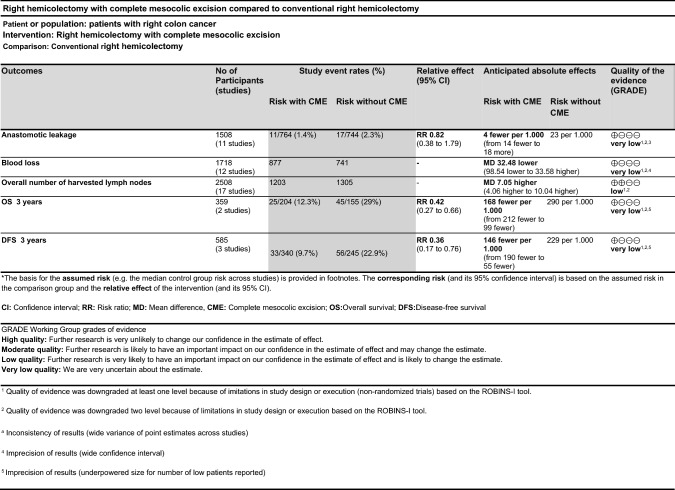
GRADE Working Group grades of evidence

For each outcome, we initially downgraded the overall quality of evidence (GRADE) by up to two levels depending on the degree of the risk-of-bias judgments and downgraded the quality further based on the GRADE evaluation criteria, as shown in Fig. 3. Overall, the quality of evidence was very low because of serious concerns regarding inconsistency and imprecision. Furthermore, other significant causes of downgrading of evidence were inconsistency of the results for the wide variance of point estimates across studies and the imprecision for the wide confidence interval of these outcomes in the few studies included. This was prominent for the more short-term outcomes, except for lymph node harvest—the only outcome reported in all included studies. The studies that reported disease-free survival (DFS) and overall survival (OS) had evidence downgraded by one level because of their imprecision; they were underpowered due to the low number of patients included. The degree of publication bias was difficult to ascertain and quantify.

Primary outcomes: statistical analyses result for secondary outcomes are presented in Table 3.

**Table 3:** Primary outcomes

	Number of studies reporting the outcome	Number of patients analysed	Occurrence in CME branch	Occurrence in non-CME branch	RR/MD	95% CI	Heterogeneity (I^2^)%
Anastomotic leak	11	1508	11/764 (1.44%)	17/744 (2.28%)	0.82	0.38–1.79	0
Blood loss	12	1618	877	741	-32.48	−98.54 to −33.58	100
Overall number of harvested lymph nodes	17	2508	1203	1305	7.05	4.06-10-04	98
3 year overall survival	2	359	204	155	0.34	0.20–0.59	0
5 year disease-free survival	3	585	340	245	0.36	0.17–0.76	61

RR (relative risk) < 1 favours CME; > 1 favours non-CME. MD (mean difference) < 0 favours CME; > 0 favours non-CME

*CME* complete mesocolic excision

### Anastomotic leak

**Fig. 4:**
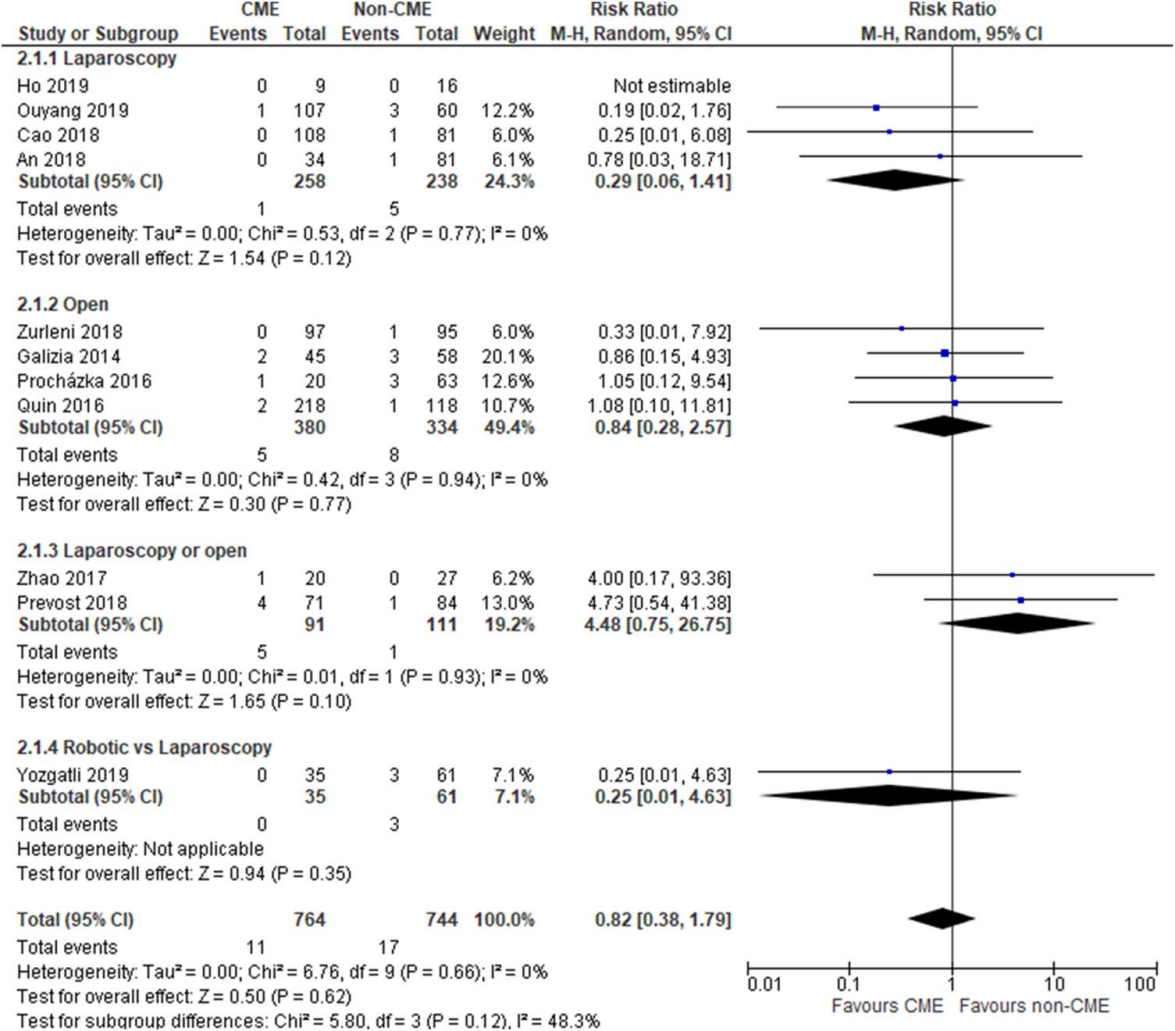
Anastomotic leak

Eleven studies [34, 37, 39, 42, 44–46, 48–51] reported this outcome (1508 patients). No statistically significant difference was found in the incidence of anastomotic leak in the CME group (1.44%, 11/764) and in the non-CME group (2.28%, 17/744), (RR 0.82, 95% CI 0.38–1.79); the same result was reported in the analysis of open and laparoscopic groups. Heterogeneity was absent (*I*^2^ = 0%) (Fig. 4). The overall quality of evidence was deemed to be very low because of multiple risk-of-bias downgrades and inconsistency of results.

### Blood loss

**Fig. 5:**
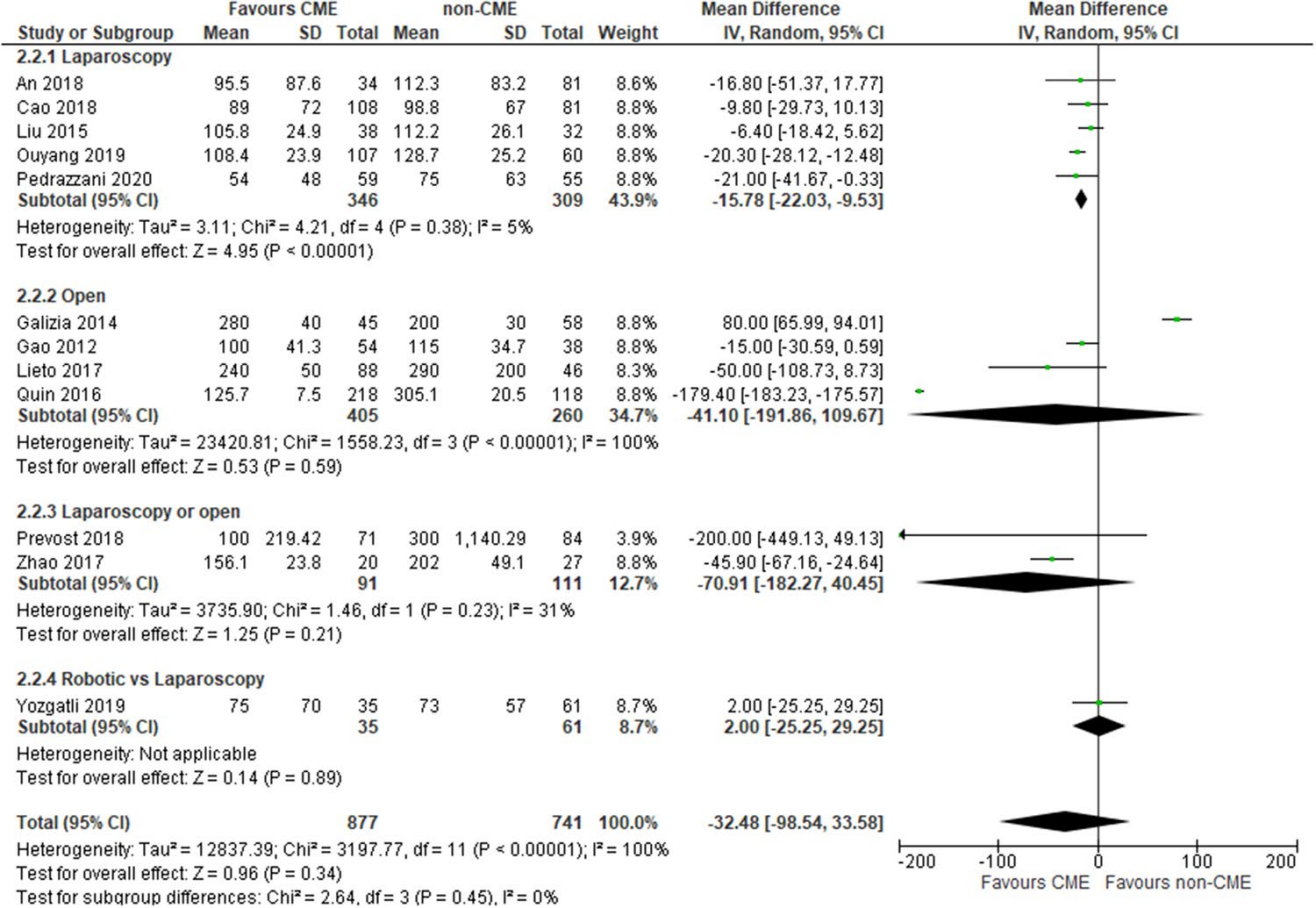
Blood loss

Twelve studies [34, 36–38, 40–44, 46, 48, 49] reported this outcome (1618 patients: 877 laparoscopic vs 741 open). There was no difference in the estimated blood loss was no different in the CME group and the non-CME group (MD −32.48, 95% CI −98.54 to −33.58), and the heterogeneity was high (*I*^2^ = 100%). Subgroup analysis reported a significantly lower estimated blood loss in the laparoscopic CME group (MD −15.78, 95% CI −22.03 to −9.53; participants = 655; studies = 5); the heterogeneity was very low (*I*^2^ = 5%) (Fig. 5). The overall quality of evidence was deemed to be very low because of multiple risk-of-bias downgrades and imprecision.

### Overall number of harvested lymph nodes

**Fig. 6:**
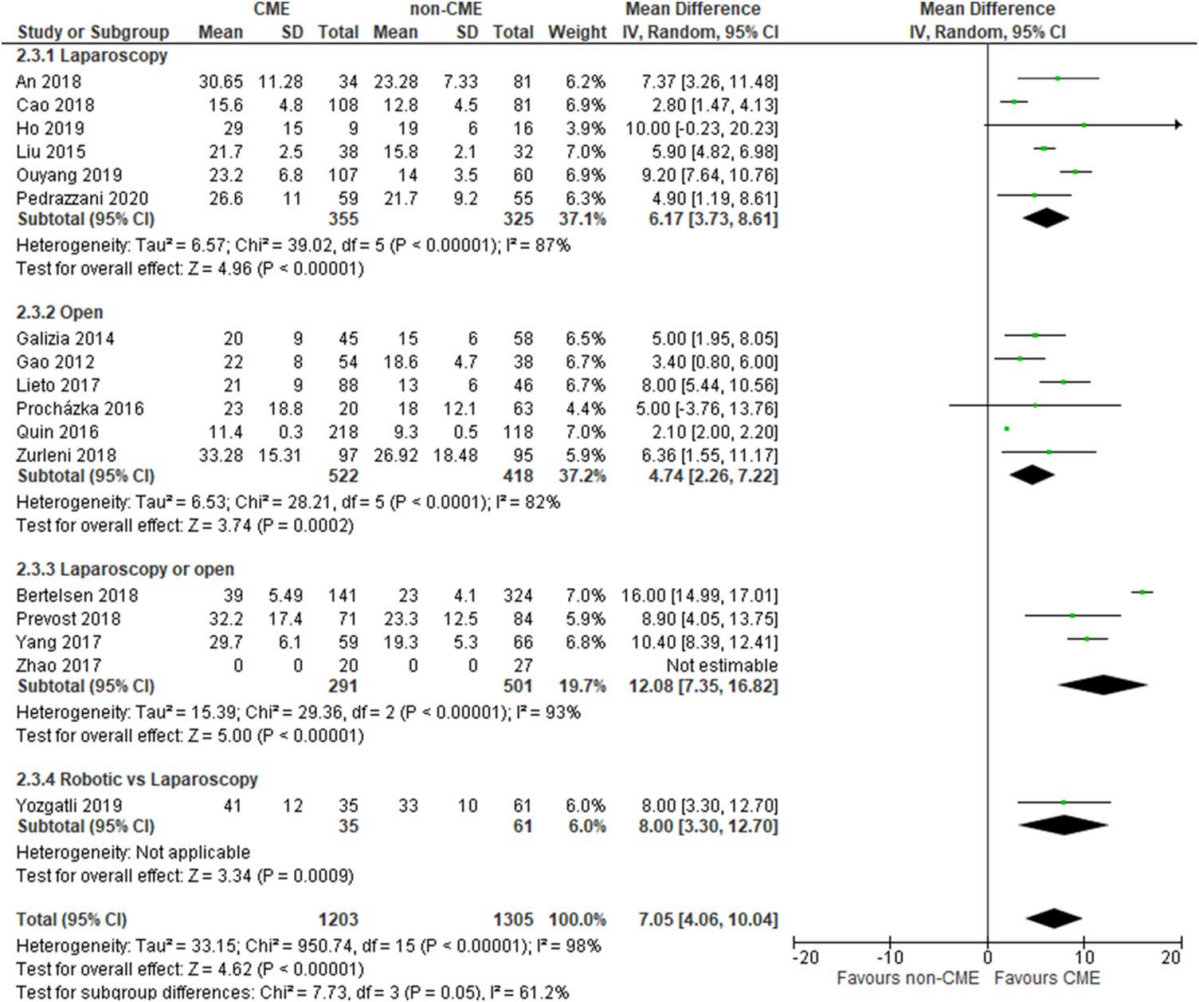
Lymph node yield

Seventeen studies [34–50] reported this outcome (2508 patients, 1203 CME vs 1305 non-CME). The overall number of harvested lymph nodes was significantly higher in the CME group (MD 7.05, 95% CI 4.06–10.04); this trend significantly favoring CME was reported in all the subgroup analyses. The heterogeneity was significantly high (*I*^2^ = 98%) and may be due to subgroup effects. Subgroup analyses of the different surgical modes of access reported a significantly higher number of harvested lymph nodes in the CME group, but the heterogeneity was very high in all the subgroups (Fig. 6). Another subgroup analysis of different populations reported that the overall number of harvested lymph nodes was statistically higher in the CME group in Asia (MD 6.16, 95% CI 3.75–8.58; participants = 1262; studies = 10; *I*^2^ = 96%) and Europe (MD 7.95, 95% CI 3.13–12.77; participants = 1246; studies = 7; *I*^2^ = 94%). Overall, the quality of evidence was deemed to be low because of multiple risk-of-bias downgrades.

### Three- and 5-year oncologic outcomes

Few studies reported the oncological outcomes at 3 and 5 years, but the data are not statistically comparable and are extremely heterogeneous between the included studies. However, all long-term oncological outcomes (local recurrence, systemic recurrence, overall survival [OS] and disease-free survival [DFS]) favored the CME vs non-CME groups (Table 4).

**Table 4:** Three-. and 5-year oncologic outcomes

Oncologic 3-year outcomes										
Author		Patients lost at follow-up	Patients evaluated	Overall recurrence	Local recurrence	Systemic recurrence	Overall survival	*P*	Disease- free survival	*P*
Ouyang [42]	CME	6	101	9 (8.4%)	3 (2.97%)	6 (5.94%)	94 (93.5%)	0.017	93 (91.6%)	0.014
	Non- CME	9	51	12 (20%)	4 (7.84%)	8 (15.6%)	43 (85.0%)		43 (85.0%)	
Zurleni [50]	CME	NR	NR	NR	NR	NR	88%	0.003	NR	< 0.01
	Non- CME	NR	NR	NR	NR	NR	71%		NR	

Oncologic 5-year outcomes										
Author		Patients lost at follow-up	Patients evaluated	Overall recurrence	Local recurrence	Systemic recurrence	Overall survival	*P*	Disease- free survival	*P*
An [34]	CME	NR	NR	NR	NR	NR	100%	0.049	94.12%	0.534
	Non- CME	NR	NR	NR	NR	NR	89.49%		89.17	
Lieto [40]	CME	NR	NR	NR	NR	NR	NR	NR	89.2%	0.02
	Non- CME	NR	NR	NR	NR	NR	NR	NR	49.1%	
Quin [46]	CME	NR	NR	NR	NR	NR	NR	NR	89.8%	0.048
	Non- CME	NR	NR	NR	NR	NR	NR	NR	82.2%	

*CME* complete mesocolic excision, *NR* not reported

**Fig. 7:**
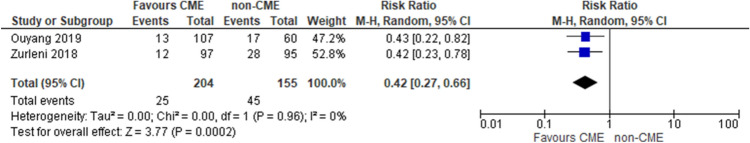
Oncological outcomes: overall survival at 3 years

Regarding 3-year oncological outcomes, Ouyang et al. and Zurleni et al. [42, 50] reported only overall survival. This was higher for the CME group in the study of Ouyang (OS 93.5% in the CME group and 85% in the non-CME group) than in the study by Zurleni (OS 88% in the CME group and 71% in non-CME group). The OS was significantly better in CME group than non-CME groups (RR 0.42, 95% CI 0.27–0.66; *p* = 0.0002. participants = 359; studies = 2; *I*^2^ = 0%) (Fig. 7).

**Fig. 8:**
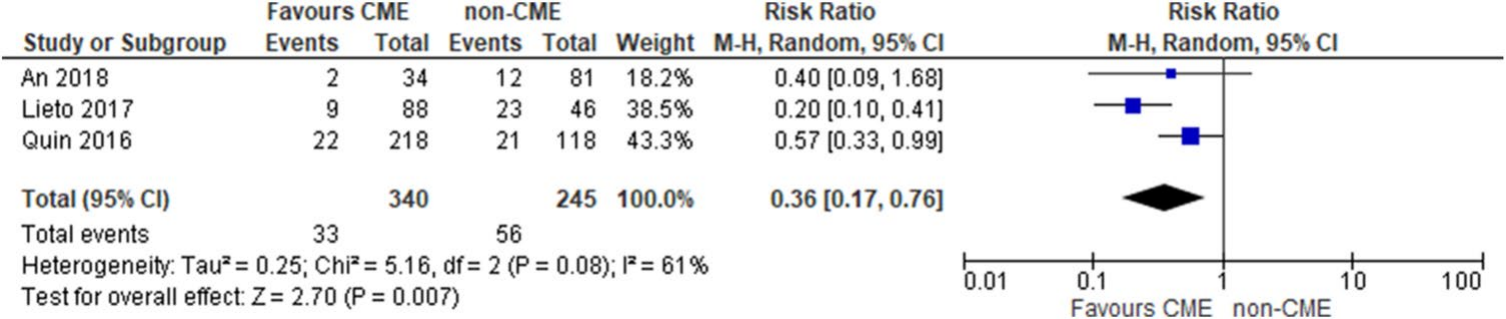
Oncological outcomes: disease-free survival at 5 years

Regarding 5-year oncological outcomes, the studies by An et al., Lieto et al. and Qin et al. [34, 40, 46] reported only DFS. This was higher for the CME group in the study of An (DFS 94.12% in the CME group and 89.17% in the non-CME group) than in the studies by Lieto (DFS 89.2% in the CME group and 49.1% in the non-CME group) and Quin (DFS 89.8% in CME group and 82.2% in non-CME group). The DFS was significantly better in CME group than non-CME groups (RR 0.36, 95% CI 0.17–0.56; *p* 0.007. participants = 585; studies = 3; *I*^2^ = 61%). This heterogeneity is probably due to more favorable tumour stage in the study of An, which reported a higher rate of patients with TNM stage I (35 patients, 30.43%), compared with a lower rate (6.25%) of patients with stage I disease in the study by Quin. In addition, there was a higher rate of patients with TNM stage IV disease (9 patients, 6.71%) in the study of Lieto compared to those of An and Quin, neither of which included patients with stage IV disease (Fig. 8). For both 3- and 5-year outcomes, the quality of evidence was deemed to be very low because of imprecision and multiple risk-of-bias downgrades.

Secondary outcomes: statistical analyses result for secondary outcomes are presented in Table 5.

**Table 5:** Secondary outcomes

	Number of studies reporting the outcome	Number of patients analysed	Occurrence in CME branch	Occurrence in non-CME branch	RR/MD*	95% CI	Heterogeneity (*I*^2^)%
Operative time	14	1736	902	834	16.43	4.27–28.60	95
Conversion from laparoscopy to open right hemicolectomy	4	682	18/212 (8.49%)	35/460 (7.6%)	1.72	1.00–2.96	0
Intraoperative complications	3	365	19/178 (10.67%)	14/187 (7.48%)	1.14	0.60–2.15	0
Reoperation rate	4	591	7/299 (2.34%)	11/292 (3.76%)	0.65	0.26–1.75	0
Overall postoperative complications	10	1307	141/786 (17.94%)	142/617 (21.16%)	0.82	0.67–1.00	0
Clavien–Dindo grade III–IV postoperative complications	3	447	19/155 (12.26%)	43/338 (11.92%)	1.36	0.82–2.28	0

RR (relative risk) < 1 favours CME; > 1 favours non-CME. MD (mean difference) < 0 favours CME; > 0 favours non-CME

*CME* complete mesocolic excision

### Operative time

Fourteen studies [34, 36–46, 48, 49] reported this outcome (1736 patients: 902 CME group vs 834 non-CME group). The authors do not report if the operative time is "calculated from skin incision to application of wound dressings", and for this reason it was judged an unclear risk of bias. The operative time (reported in minutes as mean difference, [MD]) was significantly higher in the CME group than in the non-CME group (MD 16.43, 95% CI 4.27–28.60); this trend was the same in all the subgroup analyses. The heterogeneity was high (*I*^2^ = 95%).

### Conversion from laparoscopy to open right hemicolectomy

Four studies [34–36, 39] reported this outcome (682 patients). The incidence of conversion to open from laparoscopic surgery was statistically significantly higher in the CME group (8.49%, 18/212) compared with the non-CME group (7.6%, 35/460) (RR 1.72, 95% CI 1.00–2.96). There was no heterogeneity (*I*^2^ = 0%).

### Intraoperative complications (vascular injuries or visceral perforation)

Three studies [43, 44, 48] reported this outcome (participants = 365). The incidence of intraoperative complications was the same in the CME group (10.67%, 19/178) and in the non-CME group (7.48%, 14/187), as the difference was not statistically significant (RR 1.14, 95% CI 0.60–2.15). There was no heterogeneity (*I*^2^ = 0%).

### Reoperation rate

Four studies [36, 43, 48, 50] reported this outcome (591 patients). The incidence of reoperation rate was no different in the two groups, as the difference was not statistically significant (RR 0.65, 95% CI 0.26–1.75). There was no heterogeneity (*I*^2^ = 0%). The limitation of this analysis is the lack of data about the time interval that was involved.

### Overall postoperative complications

Ten studies [36, 37, 39, 40, 42, 43, 46, 48–50] reported this outcome (1307 patients). The incidence of overall postoperative complications was found to be the same in the CME group (17.94%, 141/786) and in the non-CME group (21.16%, 142/617), since the difference was not statistically significant (RR 0.82, 95% CI 0.67–1.00 *I*^2^ = 0%).

### Clavien–Dindo grade III–IV postoperative complications

Three studies [35, 43, 48] reported this outcome (447 patients). The incidence of postoperative complications was the same in the CME group (12.26%, 19/155) and the non-CME group (11.92%, 43/338) (RR 1.36, 95% CI 0.82–2.28; *I*^2^ = 0%); these complications included anastomotic leaks and it was not possible to analyze these outcomes without including anastomotic leak.

## Discussion

The oncological principles of colon and rectal cancer surgery involve the removal of the tumour along with an adequate amount of healthy bowel, blood vessels and draining lymph nodes. TME radically changed rectal cancer surgery, bringing important improvements in terms of reduction of local recurrence [52]. In 2009 with the introduction of CME, Hohenberger[53] transferred the principles of TME to right colon surgery by demonstrating that parietal and visceral peritoneum surrounds the right colon just as the mesorectum surrounds the rectum. As for TME, following the visceral and parietal peritoneum plane, resections can be obtained along the most effective surgical planes. CME mandates surgical dissection along embryological planes with sharp separation of the visceral and partial tissue layer (in analogy to the TME concept) and true central ligation of the supplying vasculature. The aim of CME is a greater extension of lymphadenectomy, a greater volume of intact mesentery and an adequate length of bowel resection, with the hypothesis that the oncological outcome would be improved by a more radical and targeted surgical approach. The first results reported were an increase in absolute survival to 89%, compared with 81% after traditional right hemicolectomy, with local recurrence of 3.5% compared to 6.5% [53].

At a time when laparoscopic surgery for colon cancer is largely standard clinical practice and validated from an oncological perspective [54], CME is a more complex and difficult intervention, requiring more operative experience and a longer learning curve [55]. The difficulty arises primarily from the central ligation of the vessels supplying and draining the right colon. The vascularization of the right colon is extremely variable and the anatomical variations are mainly found at the level of the right colic artery and vein, the right branch of the middle colic artery and vein and in particular at the level of the venous branches of the trunk of Henle [56]. Since the oncological benefits of CME remain unclear and since laparoscopic CME is a technically difficult procedure to perform, CME has not yet become standard surgical treatment for right colon cancer.

It remains unclear whether these newer concepts of oncological radicality in colon cancer surgery need to be routinely considered, as has been the case for the appropriate use of TME in rectal cancer surgery. There is still uncertainty regarding the surgical treatment of right colon cancer. This is the basis for CME, which pursues the same oncological objective as D3 hemicolectomy, more common in Asia, or hemicolectomy with CVL [57].

Pending evidence on the oncological outcome of patients treated with CME compared with patients treated with traditional hemicolectomy for right colon cancer, the focus has largely been on short-term results and complications. The available literature is of variable quality with several retrospective, single-centre analyses providing comparisons between CME and traditional right hemicolectomy.

The strengths of our analysis include well-established guidance for conducting systematic reviews of observational and diagnostic data. We used standard pre-specified criteria for study assessment. We carefully avoided duplicate data. We performed a meta-analysis of the data, increasing sample size and precision compared to any single study. The identification of a single procedure, identified as right hemicolectomy performed exclusively to treat malignant pathology by traditional surgical approach and CME, allowed further efforts to ensure a homogenous sample, reducing the risk of bias that would have arisen when comparing different surgical procedures. It should also be noted that in most of the studies analysed, the groups of CME patients and traditional surgery patients were well distributed, without substantial differences in age, body mass index and other possible confounding factors.

The limitations of our study include the difference in sample size in the analysed studies, many of which had small numbers of patients. Most of the analysed studies are retrospective. We were able to include only three prospective studies. Only two studies had a sufficiently long follow-up to provide 5-year survival data. The variables analysed were often not homogeneously coded between the studies considered, and this reduced the number of comparable data points. An attempt at alignment of these data would have introduced possible bias. It was not always indicated whether any neoadjuvant chemotherapy was given prior to surgery, although this is not common practice. Some factors, such as the incidence of complications, were not related to the extent of the tumour according to Union for International Cancer Control (UICC) staging in any of the studies analysed. Despite these differences in the analysis of the various studies, the sample of data obtained has a much higher magnitude than that of any single study, enhancing the results of our research.

This systematic review and meta-analysis is very different from the previous ones by Ow [58] and Wang [22] in that we only included patients with right colon cancer undergoing right hemicolectomy, whereas in the other two meta-analyses, all types of colic resection were included.

For this reason, the analyses of the other two studies present a moderate heterogeneity (*I*^2^ = 60% in the analysis of 5-year overall survival and *I*^2^ = 61% in the analysis of DFS). In our meta-analysis, only a few studies reported data on distant survival, so it was not possible to perform an analysis that would provide statistically significant data.

In fact, it has been demonstrated that right colon cancer has a different disease progression and has aa worse prognosis than left colon cancer which is related to a higher number of cases of advanced disease at diagnosis [59]. It must also be taken into account that the complication rate following standard right hemicolectomy is generally higher than that following left hemicolectomy [60].

Because of the cumulative analysis of patients with different characteristics related to the site of colon cancer (right, transverse, and descending/sigma) and the multiplicity of surgical techniques performed the results of the two studies mentioned above were different from those of our study, in which the study population was homogeneous with regard to biology and surgical technique.

We divided the endpoints in our analysis into two macrocategories: one concerning the feasibility and safety of CME compared to traditional right hemicolectomy; the other concerning the oncological outcome resulting from a more radical surgical approach.

The GRADE analysis yielded a very low grade of evidence in all principal and oncological outcomes, with the exception of lymph node harvest in which the level of evidence was low. For this reason, it is not possible to draw any conclusions of CME non-inferiority and better randomized clinical control trials are needed to support the non-inferiority of CME.

As far as operating time is concerned, since the CME procedure is intrinsically more complex and detailed, it is to be expected that the duration of the operation is longer, even in the absence of a standardised parameter for measuring the actual operating room time. Although the trend shows a tendency for CME to take longer (only Lieto et al. [40] report shorter operating times for CME compared to standard hemicolectomy), this difference in our analysis was not statistically significant, and it is not associated with a greater incidence of intra- and postoperative complications, or longer hospital stay.

The conversion rate to open surgery in laparoscopic procedures was the only variable analysed that seems to favour the standard non-CME procedure, with a statistically significantly lower conversion rate. It should be noted that only four studies, with small sample sizes, have reported these data. This may be partly due to the greater technical difficulty of the procedure, in addition to the variations of the vascular anatomy of the right colon. Another aspect to consider is how a robotic approach may allow the procedure to be performed more readily than laparoscopy, and thus lead to a lower conversion rate. Early data seem to support this hypothesis, as the only robotic study we were able to evaluate [48] did not report any conversions to open surgery, or any difference in rates of complications compared to the standard procedure.

In terms of oncological radicality, CME favours lymph node yield. This may suggest oncological superiority, but unfortunately, with the studies available in the literature to date, this cannot be effectively proven by evaluating and comparing the 3- and 5-year survival in a sufficiently large sample. It has been shown that greater surgical radicality may improve the chances of long-term survival [61], but further studies, possibly randomized and with longer follow-up, are necessary to assess and quantify the real impact that this type of surgery has on the patient's oncological outcome. It should also be noted that our data show a higher heterogeneity in the lymph node yield during laparoscopic procedures if compared to open cases. This is probably due to the greater technical skill required to perform the procedure with a minimally invasive approach [62].

Our data show that it is necessary to standardize the evaluation parameters regarding complications, their treatment modalities, and their time of onset after surgery; the recording of operating time; and the standardization of parameters for evaluation of long-term survival, defining universal and easily comparable parameters, as other authors have suggested [63]. A possible confounding factor derives from the fact that, since there is no universally accepted definition that standardizes the CME surgical procedure, it is not possible to be sure that the procedures performed in the different centres have followed the same precise steps. It is necessary to have a definition of quality easily applicable to the surgical specimen obtained, outlined by univocal quantitative parameters, in order to be able to qualitatively analyse such a complex procedure.

We hope that in the near future the objective of the major surgical oncology and colorectal societies will be to plan and perform RCTs with a high level of methodological quality and adequate power analysis to clarify this important issue.

## Conclusions

With the data available to date, it is not possible to definitively demonstrate that CME has oncological superiority in terms of survival, but only that it has not proved inferior to traditional surgery in terms of feasibility and safety, and that it leads to an increased lymph node yield when compared with traditional right hemicolectomy. In the future, to identify its precise indications, its superiority will need to be proven in oncological terms, as has happened for TME in rectal cancer surgery. We are aware of five ongoing prospective studies that we will be eager to include in a future analysis and anticipate that this will allow further light to be shed in defining the role of CME in surgery for right colon cancer.
